# Bio–Microfabrication of 2D and 3D Biomimetic Gut-on-a-Chip

**DOI:** 10.3390/mi14091736

**Published:** 2023-09-04

**Authors:** Yeongseok Jang, Jinmu Jung, Jonghyun Oh

**Affiliations:** 1 Department of Mechanical Design Engineering, Jeonbuk National University, Jeonju-si 54896, Jeollabuk-do, Republic of Korea; ysjang@jbnu.ac.kr; 2 Department of Nano-Bio Mechanical System Engineering, Jeonbuk National University, Jeonju-si 54896, Jeollabuk-do, Republic of Korea

**Keywords:** intestine, microfabrication, lab-on-a-chip, gut-on-a-chip, biomimetic

## Abstract

Traditional goal of microfabrication was to limitedly construct nano- and micro-geometries on silicon or quartz wafers using various semiconductor manufacturing technologies, such as photolithography, soft lithography, etching, deposition, and so on. However, recent integration with biotechnologies has led to a wide expansion of microfabrication. In particular, many researchers studying pharmacology and pathology are very interested in producing in vitro models that mimic the actual intestine to study the effectiveness of new drug testing and interactions between organs. Various bio–microfabrication techniques have been developed while solving inherent problems when developing in vitro micromodels that mimic the real large intestine. This intensive review introduces various bio–microfabrication techniques that have been used, until recently, to realize two-dimensional and three-dimensional biomimetic experimental models. Regarding the topic of gut chips, two major review subtopics and two-dimensional and three-dimensional gut chips were employed, focusing on the membrane-based manufacturing process for two-dimensional gut chips and the scaffold-based manufacturing process for three-dimensional gut chips, respectively.

## 1. Introduction

Originally, microelectromechanical systems (MEMS) technology, as one of semiconductor processes, was employed to miniaturize electrical and mechanical systems such as sensors and actuators after the development of semiconducting fabrication technology in 1960 [1,2,3,4,5]. With the main advantages of small-scale and low-cost, it has expanded to implicate fluidic, acoustic, magnetic, optical, and biological systems [3,6,7,8,9]. The microfluidic technology and polymer chip-based microfabrication processes began to be applied to produce lab-on-a-chips in the late 1980s [10,11,12,13,14]. Over the past several decades, those technologies for lab-on-a-chip have evolved into the most popular microfabrication technologies while especially grafting those with biology and medicine more broadly [15,16,17]. Recently, advances in bio–microfabrication technologies have led to a paradigm from lab-on-a-chip to organ-on-a-chip [18,19,20,21]. This paradigm shift helps to overcome the limited functions of 2D organ tissues as well as ethical and costly problems of clinical trials in new drug development [22,23,24].

The gut, as the gastrointestinal tract, plays a significant role of facilitating digestion and absorption of nutrients in the body [25,26]. Therefore, many researchers are interested in studying defective digestive, immune, and endocrine functions as well as diseases such as infection, inflammation, and cancer using animal models. These models have been enormously challenged by unethical and costly issues caused by using different kinds of animals [27,28,29,30,31]. Therefore, a lab-on-a-chip was proposed as a solution to replace animal models with many unsolvable problems. Lab-on-a-chips are the most versatile microfluidic devices that allow total integration of multiple lab functions as well as miniaturization of functional components, such as injector, transporter, preparator, sensor, mixer, reactor, separator, controller, detector, and power supply [11,32,33,34,35,36]. These devices have been utilized in a variety of research studies targeting tissue and organ models integrated with blood vessels, heart, lung, liver, intestine, kidney, and so on [19,22,37,38,39,40,41,42,43,44,45]. To build bio-applications in the chip, different cell lines were grown in a monolayer format. A two-dimensional format has limitations in functions provided by a physiological three-dimensional structure. Thus, it cannot reflect in vivo epithelium or tissue morphology shown in a lab-on-a-chip. Although recent research has tried to shift toward three-dimensional structures for more realistic microenvironments in terms of biochemical and biomechanical aspects, two-dimensional cell culture still predominates. Many challenges in the construction of three-dimensional cell structures remain, including interface of connective tissues, mechanical microenvironment, and spatiotemporal distributions of oxygen, nutrient and metabolic wastes [20,46].

**Figure 1:**
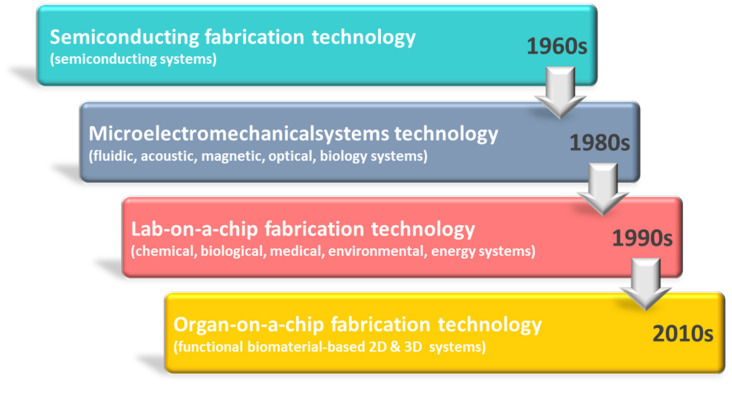
Chronological progress of primary developments in the fabrication technology of a gut-on-a-chip.

To address these challenges, specialized biomaterial-based microfabrication processes for three-dimensional structures need to be developed. With continuous progress in bio–microfabrication techniques, biomaterial-based microfabrication technology for a gut-on-a-chip has implemented a great complexity of gut tissues in vitro to mimic physiological three-dimensional structures with effective gut functions [47,48,49]. The three-dimensional structure in the gut-on-a-chip represents many intestine villi and microvilli on an enormous surface. Those micro-fabricated folded microstructures could facilitate main gut functions of harboring microorganisms for aiding digestion, immunity, and protection from foreign pathogens [50]. Topics related to lab-on-a-chip and gut-on-a-chip as mentioned above are included. Furthermore, two major review subtopics for 2D and 3D gut chips were introduced, focusing on the membrane-based manufacturing process for 2D gut chips and the scaffold-based manufacturing process for 3D gut chips, respectively. Development and recent advances made in biomaterial-based microfabrication for constructing two-dimensional and three-dimensional gut structures are comprehensively discussed in the review on an annual basis, as shown in Figure 1. In addition, one of the unique features of this review is to look at the utilization of technologies from the beginning to the present, and in the Conclusions section, we will highlight the technology that has the greatest ripple effect in 2D and 3D studies.

## 2. Microfabrication Techniques for 2D Biomimetic Gut-on-a-Chip

**Figure 2:**
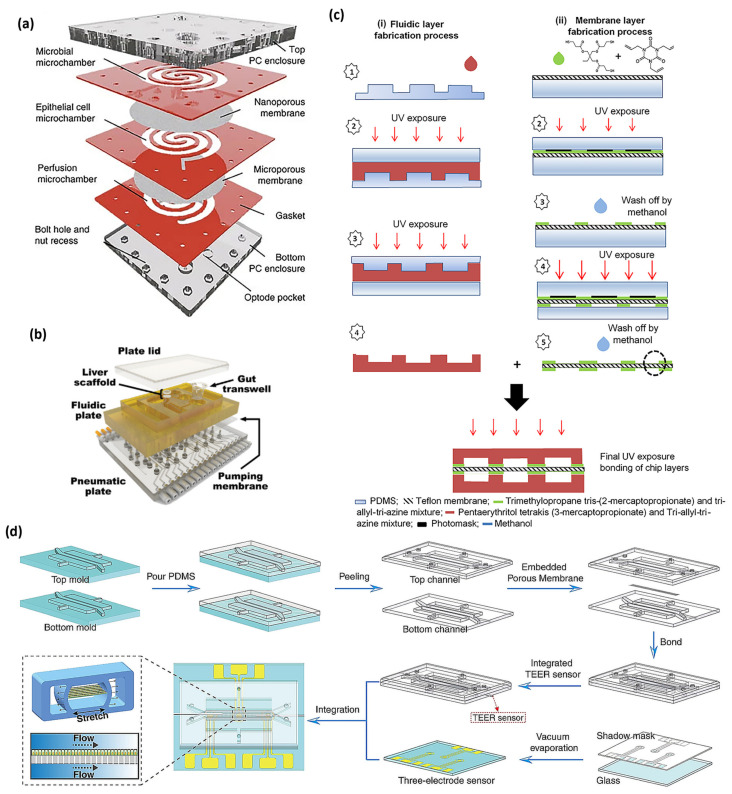
(**a**) The HuMiX device [51], (**b**) the multi-MPS platform [52], (**c**) the fabrication process of different layers of thiolene chip, containing (i) the fabrication process of upper and lower microfluidic layers and (ii) the fabrication process of the thiolene-coated teflon membrane [53], and (**d**) the production process of the gut chip integrated with sensors [54], with (**a**–**d**) being licensed under a Creative Commons Attribution 4.0 International License.

For more than a century, two-dimensional (2D) cell culture technologies have been utilized to create in vitro models for observing cellular behaviors that stimuli biophysical and biochemical signals, as shown in Figure 2. Although it has been demonstrated that three-dimensional cell culture can significantly facilitate cell proliferation, differentiation, and mechano-responses, new advances in two-dimensional cell cultured systems continue to provide new capabilities for a wide range of applications. Researchers were interested in the biomimetic chip as one of the promising applications using two-dimensional cell culture. In 2010, an in vitro living cell-based intestine model started to be developed to mimic structural, absorptive, mechanical, and pathophysiological gut properties.

The two-dimensional gut-on-a-chip was required to enable division of the microenvironment of cells or tissues in vitro and to simultaneously implement physical and biochemical functions between cells, which were necessary functions while repeating physiological functions. Researchers started to use microporous membranes to easily perform this in vitro in two dimensions. Materials used to fabricate microporous membranes are classified into categories, such as PDMS, polyester, polycarbonate, teflon, polystyrene, polyamide, and polyethylene–terephlalate. PDMS, one of the biocompatible materials, has been most widely applied using MEMS process for various 2D gut-on-a-chip applications due to the convenience of the fabrication process and the appropriateness of intestinal tissue formation [55,56,57,58,59,60,61,62,63]. The next most used material is polyester, and researchers who have difficulty in micro-processing have easily accessed 3D-printing based applications for 2D gut-on-a-chip using commercially available transwells [64,65,66,67]. As the next material, polycarbonate can be manufactured in various ways using micromachining instead of the MEMS process and has been used for various applications [51,68,69,70]. The remaining materials have been limitedly applied to some specialized gut chip applications using fabrication techniques [16,54,71,72,73]. Chronological progress of 2D gut chip bio–microfabrication is shown in Table 1.

In 2010, a micro total bioassay chip for ingested substances was introduced by Imura et al. The chip consisted of two polydimethysiloxane (PDMS) sheets on a glass slide with an upper sheet and a lower sheet, both of which had microchannels produced by photolithography [74]. Vertical microchannels were produced to connect the upper part and the lower part of the lower PDMS side [74]. The collagen-coated membrane for two-dimensional Caco-2 cell culture was placed on the microchamber. The upper PDMS sheet was laminated with the lower PDMS sheet [74].

In 2012, Kim et al. described a gut-on-a-chip with two channels separated by a porous membrane with a coating layer of extracellular matrix (ECM) for mimicking complex structure and physiology [58]. The soft lithography technique was applied to fabricate three PDMS layers with an upper layer, a porous membrane, and a lower layer permanently bonded under plasma treatment [58].

In 2014, Marzorati et al. reported the host–microbiota interaction (HMI) model as a new tool for long-term interaction study of host–microbiota in the gastrointestinal tract [16]. The mechanistic study interplay of the host–microbe was limited to reach different in vivo areas of the gastrointestinal tract (GIT) [16]. The HMI module is composed of two compartments separated by a functional double-layer consisting of an upper mucus layer and a lower permeable membrane [16]. The upper section indicates the luminal part of the GIT, while the lower section contains enterocytes indicating the host [16]. The polyamide membrane has a pore diameter of 0.2 μm and a thickness of 115 μm [16]. The mucus layer is formed by boiling distilled H_2_O including 0.8% agar and 5% porcine mucin type II and is poured onto a wet membrane [16].

In 2015, Kimura et al. developed an on-chip intestine-liver model to study pharmacokinetics [72]. The complex device being composed of a small intestine compartment, a liver compartment, and a lung compartment is connected with stir-based micropumps [72]. The small intestine compartment is separated into upper and lower sections to respond to cell polarity using a microporous membrane. It was permanently bonded between the upper and lower PDMS chips [72]. For permanent plasma bonding, a polyethylene terephthalate microporous membrane had a thin layer of aminosilane by dipping the microporous membrane into an aminosilane agent and drying the coupling agent [72].

In 2016, a human gut-on-a-chip microchip was applied to coculturing multiple commensal microbes in touch with human intestinal epithelial cells [59]. The gut-on-a-chip was made from PDMS as previously reported [59]. The PDMS gut-on-a-chip consisted of cell culture microchannel and vacuum chamber. Vacuum chambers with a width, length, and height of 1.68 mm × 9.09 mm × 0.15 mm, respectively, were the same in upper and lower parts. The microporous PDMS membrane had an array of circular holes (diameter, height, and spacing of 10 μm × 20 μm × 25 μm, respectively) [59]. Shah et al. introduced HuMiX device, a microfluidic-based in vitro interface model of the gastrointestinal human–microbe [51]. The HuMiX device consists of two polycarbonate (PC) enclosures to sandwich silicone rubber gaskets that can attach to semi-permeable PC membranes [51]. These 6.2 mm thick PC sheet enclosures were made using computer numerically controlled milling machine [51]. To describe individual spiral-shaped microchambers, silicone sheets with the thickness of 0.79 mm were trimmed to fabricate gaskets [51].

In 2017, Chen et al. developed an integrated gut/liver micro physiological system (MPS) fabricated using polysulfone and PDMS by microfabricating with on-board pneumatic microfluidic pumping [52]. In 2017, Choe et al. reported a microfluidic gut-liver chip for reproducing first-pass metabolism [64]. Soft lithography was applied for the fabrication of the gut-liver chip. When two layers of the gut and liver were bonded, channels in the gut part were facing up, whereas channels in the liver part were facing down. These two channels were placed to cross in opposite directions [64]. To assemble chips, each part was bonded on a slide glass in the order of the liver part, membrane, and the gut part from the bottom [64]. The porous membrane, which was a polyester membrane with a pore size of 0.4 μm, supported the Caco-2 cells in the top part. An additional PDMS layer was bonded with the top to hold media in the reservoir [64]. Henry et al. produced a human gut chip in organ-on-chips to measure trans-epithelial electrical resistance of human epithelial barrier function [65]. Gold electrodes were fabricated using an e-beam evaporator. After PDMS channels were defined using a CO_2_ laser with minimal power, patterned PDMS layers were formed to the size of the polycarbonate substrate to produce a PDMS channel on an acrylic “stamp” [65]. Then, the TEER Chip with integrated electrodes was assembled [65]. Trietsch et al. introduced extracellular matrix-based intestinal tubules formed in perfused microfluidic chips for showing transporter expression and tissue polarization [75]. OrganoPlate culture was conducted with three-lane OrganoPlate channels with a width and height of 400 µm and 220 µm [75]. After 2 µL of gel solution was mixed with 4 mg/mL Collagen I, 100 mM N-2-hydroxyethylpiperazine-N-2-ethane sulfonic acid (HEPES) and 3.7 mg/mL NaHCO_3_, it was distributed into the inlet and incubated for 30–45 min at 37 °C [75]. Caco-2 cells were trypsinized with 0.5% trypsin in phosphate buffered saline/ethylenediaminetetraacetic acid (PBS/EDTA), aliquoted, and pelleted [75]. Cells were supplied to the system by seeding with a density of 1 × 10^7^ of cells/mL in the outlet of the top channel [75]. Then, to enhance the cell sediment against the ECM, the plate was remained on the side for 20 min [75].

In 2018, Chen et al. suggested a pumpless body-on-a-chip with gastrointestinal (GI) and liver tissue sections connected with media flow driven by using gravity [68]. The device frame was milled out of a polycarbonate material (13 mm thick) on a computer–numerical control–milling machine [68]. The scaffold for the 3D liver tissue was placed into a cell culture chamber fabricated by sandwiching a polycarbonate membrane with a 0.4 μm pore size [68]. A nucleopore track–etch membrane with 0.4 μm pore size was placed between 2 silicone gasket rings (0.5 mm thick), considering the initial growth period in plates [68]. Fluidic channels were manually fabricated from silicone gaskets with the thickness of 0.5 mm and positioned so that media fluid could flow into each chamber from reservoir wells [68]. Guo et al. suggested modeling drug metabolism in the intestine using a biomimetic human gut-on-a-chip [71]. A master mold was fabricated using soft lithographic techniques [71]. The SU-8 mold is treated by chlorotrimethylsilane for use. The PDMS on molds was cured and delaminated from plates [71]. The unstructured support part was fabricated in the same way on a glass wafer [71]. At last, the membrane was overlapped between the channel part and unstructured support part [71]. The channel part on the glass wafer was placed to bond with the membrane using PDMS glue [71]. After stacking three layers together, the chip was glued using the PDMS solution to protect from the leakage of the medium. The whole device was then placed for curing of the PDMS [71]. Tan et al. presented thiolene ‘click chemistry’ based drug transport studies using a multi-chamber microfluidic intestinal barrier model [53]. Two sets of molds were prepared to produce fluidic parts and electrode grooves for the thiolene microchip [53]. The first master molds were fabricated using a micro-milling machine onto 5 mm PMMA blocks [53]. Second molds were made from PDMS on the PMMA master mold [53]. The top and bottom microfluidic components were produced using a mixture of tetrathiol moieties and triallyl moieties in stoichiometric ratios [53]. To fabricate the thiolene-coated teflon membrane, a commercially available teflon membrane with the pore size of 0.4 μm was modified for better bonding of the membrane to the thiolene components [53]. To finalize the bonding after aligning the layers, combined layers were placed under UV radiation on each side for an additional minute [53]. Bottom and top electrodes were fabricated using indium and platinum wire [53].

In 2019, Ramme et al. cultivated models of four human organs consisting of intestine, liver, brain, and kidney in a microchip, which is called an autologous induced pluripotent stem cell-derived four-organ-chip [69]. Soft lithographic and replica molding techniques using PDMS were used for the fabrication of the chip [69]. One master mold was produced by aluminum milling for the surrogate blood circuit and the other was fabricated for the excretory circuit [69]. A polycarbonate adapter plate that was 10 mm thick was treated using a silicon rubber additive and fixed to the master mold to generate the excretory circuit. The insertion of screws into the holes of the adapter plate was completed to form PDMS-free culture compartments and PDMS membranes with the two on-chip micropumps [69]. The first PDMS slice generated fluid-tight bonding of the adapter plate and the second PDMS slice on the bottom blood circuit was placed without an adapter plate [69]. A polycarbonate membrane with pore size of 1.0 μm was under plasma treatment and was put in 1% (3-Aminopropyl) triethoxysilane aqueous solution for 20 min at 20 °C [69]. The dried membrane was put on the space supplied on the bottom PDMS at the location of the glomerulus and tubules parts [69]. To prohibit the bonding of PDMS membranes, they were drawn upward under a vacuum of less than −20 kPa [69]. Sakharov et al. provided a systems biology study on comparing Caco-2 cells cultured on a conventional 2D surface and in a microfluidic chip [76]. The microfluidic chip was composed of three layers; the first layer was a 10 mm polycarbonate plate with wells functioned by lids and ports. The second was a 2 mm PDMS layer with the microfluidic circuit and wells, and the last layer was a glass slide [76]. The well contained a unit of HTS Transwell^®^-96 well permeable support with two-dimensional Caco-2 monolayers [76]. Shin et al. produced a longitudinal co-culture chip for anaerobic gut microbiome interface [62]. The soft lithography method was applied to the chip, as described previously [58]. Upper and lower microchannel layers were fabricated by demolding from a 3D printed cast and curing PDMS [62]. A microporous PDMS membrane was produced with PDMS on a silicon wafer to convey an array of micro-pillars with the diameter, height, and spacing of 10 µm, 20 µm, and 25 µm [62], respectively. The PDMS prepolymer solution was poured on the wafer and then it was spread with a thin fluoropolymer-coated polyester film, pressed using a 3 kg weight, and cured in an 80 °C oven for 12 h [62]. The upper PDMS layer and PDMS membrane were bonded together under plasma treatment and then bonded to the lower PDMS layer by oxygen plasma treatment after alignment under a stereomicroscope [62]. Lastly, a bent connector connected with silicone tubing was inserted into each microchannel for supply of oxic or anoxic cell culture medium and application of vacuum suction [62].

In 2020, Jing et al. studied host–microbial interaction using peristaltic human gut-vessel microsystem [57]. PDMS plates were fabricated using photolithographic and soft lithographic techniques [57]. All microchannels were generated by casting PDMS prepolymer solution-coated on a mold micro-fabricated with an inverse channel design [57]. After peeling of microchannel layers from the wafer, peripheral holes for tubing were produced on PDMS plates [57]. Additionally, holes with 5 mm in diameter were produced in the center of each PDMS plate for the facilitation of connections between intestinal epithelial cells and the endothelial monolayer [57]. Porous PDMS membranes were fabricated by casting PDMS prepolymer on a wafer having microarray with circular pillars with the diameter, height, and spacing of 10 μm × 20 μm × 25 μm [57], respectively. Porous PDMS membranes with diameter of 5 mm and pore size of 10 µm were put between plates for on-chip cell culture [57]. PDMS plates were assembled with top and bottom polymethyl methacrylate (PMMA) frames [57]. Langerak et al. reported a novel 3D-printed microfluidic chip fabricated using an inert biocompatible polylactic acid to gain minimization of solute absorption for optimized cell differentiation [77]. The device was fabricated by fused deposition modeling 3D printing [77]. The device was generated using a 0.4 mm nozzle and a layer resolution of 60 microns. In the printed devices, top and bottom were partially open and sealed with cover slips for easy imaging [77]. In a typical chamber with length, width, and height of 75 mm, 12.5 mm, and 3.65 mm, respectively, an open reservoir with a dimension of 25.1 mm (length) and 3 mm (width) sits in the center of the chamber [77]. Two flow pathways consisting of tubular channel with 1.47 mm in diameter and cuboid channel with 1.3 mm wide and 0.7 mm long, are left by 0.4 mm space along the thickness direction of the chamber [77]. Sunuwar et al. have demonstrated that mechanical stimuli can control heat-stable enterotoxin–cycle GMP signaling of *Escherichia coli* in a human intestine–chip model [63]. The top (1 mm wide and 1 mm high) and bottom (1 mm wide and 0.2 mm high) channels of the intestine–chip were separated by a 50 µm thick PDMS membrane with 7 µm in pore diameter and 40 µm spacing, and they were surrounded on both sides by two vacuum chambers with 1 mm in height and 300µm in width [63]. These chips were coated with a solution of collagen IV and incubated in a 37 °C incubator for 2 h [63]. Enteroids, as organoids derived from small intestine, were isolated from Matrigel treated with a cell recovery solution at 4 °C for 30 min. They were dissociated with recombinant enzymes for enteroid fragments [63]. Approximately 200 fragments/chip were applied to the upper channel of the chip and incubated overnight at 37 °C [63]. Yuan et al. introduced a gut-on-a-chip system using optical coherence tomography for visualized cellular layers and bacterial colonization [73]. To create the ability to replicate the top and bottom channels of their own design, the master mold was photolithographically fabricated for a 150 μm-thick patterned SU-8 layer on a flat glass wafer [73]. These channels had a width, length, and height of 1 mm, 1 cm, and 150 μm [73], respectively. A polytetrafluoroethylene (PTFE) membrane with a 0.4 μm pore size was coated with 30 μm thick collagen and placed between upper and lower channels [73]. The membrane was treated with plasma and placed on the lower channel. The upper channel was aligned with the lower PDMS channel under a microscope [73]. Aligned PDMS channels were pressed and bonded to each other [73].

In 2021, Bossink et al. suggested organ-on-chips with cleanroom-free assembly of multiplexable electrodes for the measurement of barrier function [55]. To fabricate top and bottom channels, two PMMA molds were designed and micro-milled [55]. The PDMS channels were formed on the two PMMA molds and cured for 4 h at 60 °C [55]. Holes on inlets and outlets of the PDMS top layer were made using a 1 mm biopsy puncher [55]. A PDMS membrane with 2 μm in thickness and 5 μm in pore size was produced using the previously developed protocol [55]. An array of columns was fabricated using two methods of photolithography and soft lithography. A solution of PDMS mixed with hexane was spin-coated and cured on the master mold with photoresist columns [55]. After the photoresist was removed using acetone, the released membrane was permanently bonded to the PDMS top layer using oxygen plasma treatment [55]. The PDMS membrane was separated from the inlets of the bottom channel [55]. Next, the bottom layer was treated with oxygen plasma and bonded to the membrane and top layer [55]. Kim et al. have developed a gut–brain axis-on-a-chip to study transport across endothelial and epithelial barriers [66]. Master molds were directly 3D printed using an Anycubic Photon LCD printer. These molds were coated with trichloro (1H,1H,2H,2H-perfluorooctyl) silane as a coupling agent [66]. Then, this coupling agent was incubated with molds in a vacuum overnight [66]. After cured PMDS parts were assembled, the polyester membrane for the cell culture area was separated from transwell inserts with 0.4 µm in pore size [66]. Nelson et al. reported a human gut-on-a-chip that could perform an engineered live bacterial therapeutic for treating phenylketonuria [60]. The microfluidic chip was fabricated from PDMS material using soft lithography. It had two compartments separated by a thin, flexible, porous membrane 50 µm in thickness, 7 µm in diameter (for pores), and 40 µm in space [60,61].

In 2022, Chin et al. generated a mucus layer on a gut cell layer in a 2D gut chip for in vivo phage applications [78]. PDMS material was used to fabricate gut-on-a-chip devices using soft lithography techniques. The 3.0 × 10^5^ HT29-MTX-E12 tumorigenic goblet cells were seeded in each device which was maintained in Dulbecco’s modified Eagle’s medium under flow conditions at a flow rate of 120 μL/h [78]. Jeon et al. have studied the contribution of the microbiome to intestinal inflammation using a gut-a-on-a-chip with microelectrode arrays [79]. The gut-on-a-chip master mold was fabricated using a SU-8 photoresist on a silicon wafer using photolithographic technology. A positive photoresist was photolithographically patterned on a glass wafer by exposure to UV light through a mask. Chromium and gold were patterned onto the glass wafer to have electrodes with a thickness of 5 nm and 50 nm by an e-beam evaporation [79]. After magnetron sputtering, the unexposed part of the photoresist was removed to generate a metal electrode pattern [79]. The PDMS gut-on-a-chip mold was prepared with a 10:1 mixture rate of a silicone elastomer and curing agent and was treated under oxygen plasma to bond with microelectrode array substrates [79]. Zhao et al. have explored host–microorganisms using an embedded membrane microfluidic chip for the human intestinal–vascular microsystem [70]. Microfluidic devices of gut-on-a-chip used included PC porous membranes, a sealing channel, and cell microculture chambers utilizing a soft lithography technique [70]. Membranes with 8 μm in pore size were served as the middle layer between the two pieces of PDMS, including channels [70]. A membrane with 400 μm in diameter was gently sandwiched between the sealing channels with 500 μm in diameter [70].

In 2023, Haan et al. fabricated membranes with micropores using re-useable SU-8 molds for organs-on-chips [56]. A chromium-on-glass photomask for the photolithography process was designed using CleWin to include a square array of holes with diameter, pitch, and footprint of 12 μm, 100 μm, 25 mm × 25 mm [56], respectively. Each array included 250 × 250 holes for a total of 62,500 holes. SU-8 molds for the formation of porous membranes were photolithographically fabricated [56]. These thin, porous PDMS membranes were then replicated from the SU-8 master mold via soft lithography [56]. The mold used for the formation of membranes and was cleaned with isopropanol and oxygen plasma (29 W, 30 s) and then resilanized with Trichloro (1H,1H,2H,2H-perfluorooctyl) silane (PFOCTS) [56]. Torn-off PDMS pieces of membranes could be dissipated by coating them with an excess of the PDMS prepolymer cured at 70 °C for 2 h, and by peeling off the cured PDMS with PDMS pieces embedded in the membranes [56]. Lee et al. have fabricated a gut–mucus chip for an intestinal absorption study [67]. A SU-8 mold with channels was photolithographically produced for the realization of channels of the gut–mucus chip [67]. PFOCTS was used on the finished wafer mold to remove the cured PDMS easily [67]. The PDMS channel layer was 3 mm thick in one channel layer and 1 mm thick in the other channel layer [67]. The membrane was separated from a trans 6-well with 0.4 μm in pore diameter. Chips were assembled using plasma treatment [67]. Wang et al. have explored the transport mechanism of Hg (II) using a gut-on-a-chip fabricated by PDMS using soft lithography [54]. The chip is composed of three parts: top and bottom layers with microchannels (2.0 mm in width × 0.25 mm in height) and the middle porous membrane (20 μm in thickness; 5 μm in diameter of the pore) [54]. The porous membrane is placed between the top and bottom layers, playing an important role in forming the tissue interface [54]. The top layer, porous membrane, and bottom layer were bonded together using oxygen plasma [54]. Finally, a thin stainless-steel tube with the internal diameter of 0.8 mm was utilized for connection of PDMS microchannels using a silica gel capillary [54].

In the above, the manufacturing processes of 2D gut chips were reviewed from 2010 to 2023. Most fabrication processes involved commercialized or self-made porous membranes. These ECM-coated porous membranes are thought to have been widely used for the convenience of manufacturing gut chips. This convenience seems to have served as a limiting factor in mimicking the biological characteristics of the gut. Nevertheless, until recently, membrane-based fabrication processes were used for 2D guts.

## 3. Microfabrication Techniques for 3D Biomimetic Gut-on-a-Chip

**Figure 3:**
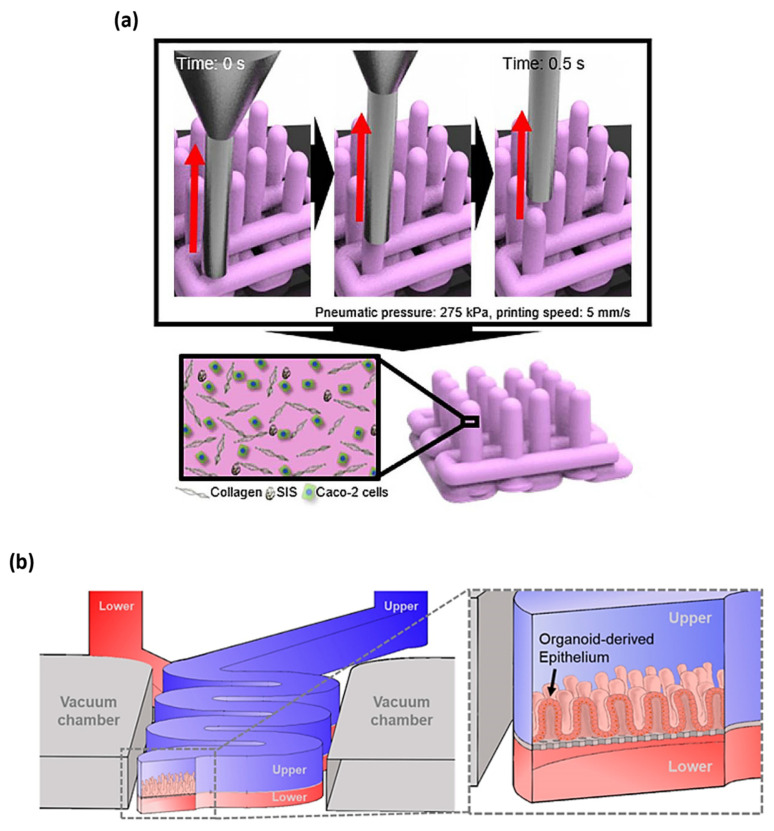
(**a**) The 3D printing technology using two bio-inks of collagen and SIS ink for establishing Caco-2-laden 3D intestinal architecture [80], (**b**) the 3D schematic of upper and lower microchannels biomimicking the lumen and capillary of the gut [81], with (**a**,**b**) being licensed under a Creative Commons Attribution 4.0 International License.

Although these 2D approaches have been well accepted in general and have significantly advanced our understanding of the normal behavior of a cell, growing evidence now show that (under some environments) 2D cell systems can lead cell bioactivities to deviate in vivo responses. For example, some main characteristics of cancer cells could not be appropriately modeled in the 2D culture environment. To overcome the limitation to their similarity with native conditions, innovative 3D cell culture environments were created to better mimic in vivo conditions, as shown in Figure 3. The chronological progress of the 3D gut chip bio–microfabrication is shown in Table 2.

In 2013, Kim et al. induced human intestinal cells to go through villus differentiation in a gut-on-a-chip [82]. Using both technologies of photolithography and soft lithography, the upper and lower microchannel parts of the gut chip were prepared by demolding cured PDMS from a SU-8 mold with a positive patterned structure made of photoresist [82]. The porous PDMS membrane was produced by casting PDMS prepolymer solution onto a master mold including post arrays of circular pillars with 10 mm in diameter, 20 mm in height, and 25 mm in spacing and pressing a silanized PDMS slab with 3 kg weight on the experimental setup [82]. The cured PDMS membrane was separated from the PDMS slab. The membrane was then attached to an upper PDMS part following an oxygen plasma treatment on the surface of each part [82]. The exposed bottom surface of the porous membrane-upper part structure was permanently treated with oxygen plasma; aligned under a microscope with the surface of the bottom part with plasma treatment [82]. The membrane surface was coated using a mixture of extracellular matrix (ECM) proteins consisting of collagen and Matrigel before cell seeding [82]. To gain a confluent cell monolayer, cells with 1.5 × 10^5^ cells cm^−2^ were plated on the upper surface of the ECM-coated porous membrane and cultured for 3 days under fixed flow of the culture medium with 30 mL h^−1^ for a shear stress of 0.02 dyne cm^−2^ through both of upper and lower channels under cyclic mechanical strain with control values of 10% and 0.15 Hz for mechanical mimicking of the active intestine microenvironment [82].

In 2015, Maschmeyer et al. suggested a four-organ-chip (4OC) for the interconnection of liver, human intestine, kidney, and skin using pulsatile fluid flow for long-term co-culture [40]. Two upper small and lower large PDMS layers included channels, membranes, micropumps, and openings for culture compartments, respectively [40]. The larger layer and a glass microscope slide were permanently bonded using low pressure oxygen plasma [40]. Then, a polyester track etched (PETE) membrane with the diameter of 4 mm and the pore size of 1 μm was glued to the PDMS layer [40]. Furthermore, the first layer and the second PDMS layer was permanently bonded together using low pressure oxygen plasma [40]. EpiIntestinal™ models were produced at MatTek Corporation [40]. Cell culture inserts were directly transferred to their respective parts of the 4OC systems [40]. The 3D barrier models were located about 100 μm above the slide glass with each 4OC circuit to confirm free media passage in the 3D intestinal barrier models [40]. Kim et al. have contributed to the study on mechanical deformation for inflammation and overgrowth of an intestine in a human gut chip [59]. The gut chip was fabricated using PDMS material as reported previously [59]. A flexible, thin extracellular matrix-coated PDMS membrane including an array of pores with a diameter of 10 μm was lined using human Caco-2 cells [59]. Cells were exposed to a flow condition of 30 μL/h, equivalent to 0.02 dyne/cm^2^ shear stress through upper and lower PDMS microchannels and to cyclic peristalsis-like mechanical deformations with 10% in cell strain and 0.15 Hz in frequency using cyclic controlled suction to hollow side chambers [59].

In 2017, Shim et al. produced a microfluidic gut-on-a-chip with 3D villi structure using collagen scaffolds [83]. Three layers were fabricated from PDMS using soft lithography and were bonded with 3D collagen villi located on the PET membrane with plasma treatment [83]. In order to fabricate the 3D villi structure, a wafer mold with an inverse structure of the villi was produced using photolithography [83]. A villus arrayed SU-8 mold was replicated from the master mold and permanently bonded to a gasket for fabrication of an alginate inverse mold [83]. Lastly, a villi-shaped collagen scaffold was fabricated from the alginate replica mold, which was dissolved. The villi-shaped collagen structure was glued to a porous membrane support fixed inside the PDMS chip with collagen [83]. Villenave et al. have introduced a human gut chip to study polarized infection of Coxsackie B1 virus in vitro [84]. Gut chip devices including two hollow microchannels with the size of 1 mm wide × 200 μm tall × 1.4 cm long were separated by a porous membrane with 10 μm in diameter (circular pore wand) and 25 μm in spacing and were fabricated from PDMS using soft lithography techniques, as previously reported [82]. Human Caco-2 intestinal epithelial cells for villus epithelium were cultured for 6 days in a gut chip device under apical flow with 30 μL/h and 0.02 dyne cm^−2^ as well as cyclic mechanical strain with 10% at 0.15 Hz [84]. Wang et al. have developed a micro-engineered collagen scaffold to form a polarized crypt-villus structure using human small intestinal epithelium. PDMS stamp #2 was trimmed for a size of 6 mm × 6 mm and coated using poly (ethylene glycol) to remove collagen adhesion after molding [85]. These PDMS stamps were coated with poly (ethylene glycol) and then polymerized using UV and were placed in a glass tube with a screwcap [85]. The tube was filled with a mixture of 10 wt% poly (ethylene glycol) methyl ether acrylate monomer, 0.5 mM sodium periodate, and 0.5 wt% benzyl alcohol in water and then exposed to UV radiation for 4 h [85]. Stamps were rinsed using deionized water and soaked in deionized water overnight to eliminate polymer and monomer residues [85]. These grafted stamps were kept in 75% ethanol until use [85]. A collagen-based scaffold was micro-molded and cross-linked on the surface of a porous polytetrafluroethylene membrane with pore size of 0.4 mm in a 12-well insert [85].

In 2018, Gregorio et al. fabricated a micro-patterned endogenous stroma equivalent that could induce a polarized crypt-villus structure of human intestinal epithelium [86]. The 3D intestinal stromal equivalents were fabricated by transferring human intestine microtissue precursors into an assembling chamber which contained a silicon mold with disc-shaped space where biological assembling of human intestine microtissue precursors took place [86]. To obtain a disk-shaped 3D enteric interstitial equivalent with a patterned surface, the top of the silicon mold was demarcated by a polymethylmethacrylate hold grid machined with a micro-milling machine. [86]. The PMMA mold represents this structure with an outer periphery of 48 mm in diameter supplied by four 5 mm diameter circles to accommodate screws, and an inner periphery of 36 mm in diameter supplied with an array of holes [86]. This holed grid generates a specific hole density of 34.1 holes/mm^2^ [86]. The basolateral chamber was full of 600 μL of DMEM to promote horizontal spreading of Caco-2 throughout the culture in water. The basolateral chamber was full of 600 µL of DMEM to perform a culture that promoted Caco-2 horizontal spreading [86]. In order to induce differentiation of 3D epithelial tissue, we performed an optimized air–liquid interface culture for 2 weeks [86]. Wang et al. have formed human colonic crypt array to apply for chemical gradients across a shaped epithelial monolayer [87]. A PDMS stamp was used to micro-mold a cross-linked collagen scaffold with a microwell structure on top of the porous membrane of a modified 12-well transwell insert [87]. The modified insert was constructed by removing the polycarbonate porous membrane from the transwell insert using sandpaper [87]. A hydrophilic PTFE porous membrane was attached to the insert utilizing a biocompatible transfer adhesive [87]. An impervious cyclic olefin copolymer plastic film was attached to block the back side of the porous membrane to reduce the effective area of the porous membrane [87]. A collagen mixture was added to the center of the transwell insert and a PDMS stamp was put on the top of the collagen mixture [87]. The PDMS stamp was demolded from the solidified collagen scaffold [87]. Workman et al. have introduced an intestine–chip that combines strengths of both human induced pluripotent stem cells (iPSCs) -derived intestinal organoids and small microengineering technologies [88]. Then, the PDMS prepolymer solution was cast onto molds to form microchannels of the upper layer and lower layer [88]. The membrane was produced from a silicon mold fabricated using two technologies of photolithography and deep reactive ion etching induced to 7 mm pores [88]. The upper part, membrane, and lower part were bonded using plasma bonding for the formation of a complete chip [88]. iPSCs were directed to produce definitive endoderm, epithelial structures, and to ultimately produce organoids [88]. Dissociated intestinal epithelial cells or Caco-2 cells were seeded into the chip to form a 3D intestine structure [88].

In 2019, Castano et al. produced a villi-like hydrogel (PEGDA and acrylic acid) scaffold with a moldless method for generation of a 3D intestinal tissue model [89]. A chip consisting of black-coated polystyrene supports and a 1 mm thick PDMS stencil containing pool arrays was utilized for hydrogel UV polymerization [89]. Villi-like micropillars have been produced using patterned photomasks on glass coverslips or porous membranes [89]. The photomask was patterned with an array of circular UV transmission windows (radius 50 μm) at a density of 25 windows mm^−2^ [89]. Pattern dimensions were chosen to match structures found in small intestine tissue to mimic the small intestine [89]. Prepolymer solutions were exposed from 60 to 220 s under UV exposure to form micropillars [89]. Samples were contained in PBS at 4 °C for at least 3 days for equilibrium swelling [89]. After reaching equilibrium swelling, cross-sections of micro-structured hydrogels were accomplished using a scalpel [89]. The cells were seeded on the hydrogels coated on coverslips at a density of 5 × 10^5^ cells cm^−2^ or hydrogels coated on inserts fabricated at densities of 1.5 × 10^5^ or 2.5 × 10^5^ cells cm^−2^ for 2D discs or 3D microstructures [89].

In 2020, Kim et al. introduced an intestinal model with villi structure produced utilizing a collagen/SIS-based cell-laden bioink and bioprinting process to perform efficient digestion and absorption [80]. The Caco-2-embedded 3D intestinal villi with collagen/SIS bioink (CLIV-CS) were produced using a 3D printing system [80]. To obtain 3D intestinal villi containing an epithelium layer and capillaries, the formation of 3D intestinal villi with an epithelium layer and capillaries was performed by two bioinks of Caco-2-laden collagen/SIS bioink (as shell bioink) and human umbilical vein endothelial cell laden collagen/SIS bioink (as core bioink) [80]. To print each villus structure, pneumatic pressures were concurrently applied for the core region using core bioink and the shell region using shell bioink [80]. Shin et al. have three-dimensionally regenerated the patient-derived intestinal organoid epithelium on a biomechanical mucosal interface-on-chip [81]. The device was seeded with Caco-2 and organoid-derived epithelial cells after the surfaces of the convoluted microchannels were activated and coated with extracellular matrix (ECM) proteins [81]. Microchannels were full of an ECM including 1% (*v*/*v*) Matrigel and 30 µg/mL of collagen I, incubated in a CO_2_ incubator for 2 h, rinsed out using medium at 100 µL/h for 1 h via a syringe pump and then used to seed dissociated organoid cells and Caco-2 [81]. The organoid culture medium was perfused to upper and lower microchannels at 50 µL/h and cyclic mechanical strain using two values of 5% in cell strain, and a 0.15 Hz in frequency was applied for the induction of 3D morphogenesis [81].

In 2021, Fois et al. investigated shear stress and dynamic flow for morphology and polarization of intestinal cells in an organ-on-a-chip model [90]. The gut chip was produced as a simple chamber device with inlet and outlet using soft lithography techniques [90]. Prior to seeding Caco-2 cells, the chip was coated with Matrigel at a final concentration (1:30) in serum-free F12 media and incubated at 37 °C for 1 h [90]. To test the effect of shear stress for cell polarization, the flow rate was fixed across the experiment at 29 µL/h, or the flow was reduced to 18 µL/h between day 5 and day 8 [90].

In 2022, Rudolph et al. produced a crypt-villus scaffold structure to bioengineer functional human intestinal epithelium [91]. The 3D printing-based resin molds were utilized to fabricate reverse molds in PDMS for the creation of silk scaffolds [91]. Thin film coated using silk fibroin was formed on the surface of PDMS reverse molds [91]. After these molds full of silk solution were frozen overnight at −20 °C, they were moved into a lyophilizer [91]. Dried spongy silk scaffolds were autoclaved to form β-sheets [91]. Then, scaffolds were poured in distilled water for 24 h to separate from the PDMS molds easily and to trim them into a rectangle, with the luminal size of 4 mm diameter and length of 8 mm, using an indented lumen in the center [91]. The fabrication process induced a half-scaffold consisting of a patterned lumen and a porous bulk part [91]. Caco-2/HT29-MTX cells, according to a 3:1 ratio and a density of 1 × 10^6^ cells/mL, were seeded on the lumen with villi and crypts of 3D half scaffolds, while the bulk part with interconnected pores was seeded with InMyoFibs on a collagen gel [91]. Shin et al. have generated 3D in vitro morphogenesis using human intestinal epithelium in a gut chip or a hybrid chip installed with a cell culture insert [92]. Both the gut chip and hybrid chip were produced by PDMS replicas demolded from silicon molds micro-patterned with SU-8 photoresist using soft lithography [92]. Microchannels in each chip were designed by considering fluid dynamic factors such as hydrodynamic pressure and shear stress [92]. The design of the gut chip includes two parallel straight-lined microchannels placed in proximity to each other. It has evolved into a complex field-on-a-chip containing a pair of curved microchannels to induce conditions, such as increased fluid residence time, nonlinear flow patterns, and multiaxial deformation of cultured cells. To induce 3D morphogenesis, linear and convoluted gut chip designs were fully interchangeable [92]. The PDMS replica cured on the master molds, with SU-8 photoresist patterns, supplied a negative feature once they were demolded [92]. To produce a gut chip, an upper PDMS layer was bonded with a porous PDMS membrane and consecutively aligned to the lower PDMS layer by conducting permanent bonding through the action of oxygen plasma [92]. To produce a hybrid chip, a PDMS replica was bonded with a glass slide to form a single-channel microfluidic device able to hold a transwell insert [92]. The functionalized PDMS surface was coated with ECM proteins and followed by the induction of dissociated organoid epithelium [92]. After the cell attachment on the functionalized PDMS surface, microfluidic cell culture started perfusing the culture medium to the upper microchannel until cells developed an intact monolayer maintaining the lower microchannel under static conditions [92].

In 2023, Wang et al. reported a gut chip to investigate the transport mechanism of Hg (II) [54]. The gut chip was produced using PDMS material [54]. The chip was composed of three compartments: top and bottom compartments with microchannels with 2.0 mm width × 0.25 mm height and the middle porous membrane with 20 μm in thickness and 5 μm in pore diameter [54]. The top part, porous membrane, and bottom part were bonded together using plasma treatment [54]. Two factors of flow rates and mechanical stretch were controlled via a high-precision syringe pump to form 3D villi-like structures [54]. Liu et al. have established a gut chip with a function of controllable oxygen gradients to investigate the effect of *Bifidobacterium bifidum* for inflammatory bowel disease [93]. The human gut chip was produced using the soft lithography method with PDMS material [93]. Two microchannels fabricated in corresponding layers had dimensions of 1 mm × 10 mm × 0.5 mm and 1 mm × 10 mm × 0.2 mm (width × length × height), respectively, and had the same size of vacuum chambers [93]. The porous PDMS membrane was produced by casting a PDMS prepolymer solution onto a micro-patterned silicon master wafer including post arrays of circular pillars with 10 μm diameter × 20 μm height and 25 μm spacing [93]. The membrane was permanently bonded between upper and lower layers using corona plasma treatment [93]. Then, the porous membrane surface was coated with collagen I and Matrigel for 1 h [93]. Human Caco-2 cells were seeded into the upper microchannel and incubated to help cells to attach to the collagen I and Matrigel-coated membrane under a static condition [93]. After 1 h of cell attachment, the culture medium was perfused constantly through the upper channel at 35 μL h^−1^ under a fluid shear stress of 0.02 dyne cm^−2^ on the first day of cell culture to create an intact monolayer [93]. Thereafter, the medium was flowed into both upper and lower microchannels at the same rate with a cyclic mechanical strain of 10% and 0.15 Hz [93].

Above, the manufacturing process of 3D gut chips was summarized and reviewed from 2013 to the present. To overcome the biological limitations of the porous membrane-based 2D gut chip reviewed in Section 2, 3D villi were designed for precise simulation of the gut. To manufacture it, various processes such as bio-printing, micromachining, stamping, and replica molding were utilized to take a step closer to the biological simulation of gut villi. However, this tended to reduce utilization by increasing the complexity of the fabrication process.

## 4. Conclusions

Various experiments or simulations have been performed to overcome challenging problems arising from studies related to intestinal diseases. After 2010, a clue to solve these problems was found in an in vitro intestine model. With semiconductor fabrication technologies as s starting point, different types of bio–microfabrication technologies have been developed due to the interest in producing in vitro 2D and 3D functional intestine models on chips. The flexibility of an intestine chip makes it possible to study factors affecting intestinal diseases and their interactions intensively.

A 2D intestine chip was mainly used as a base for growing cells by applying a porous micromembrane, providing convenience in fabrication. The flexibility of an intestine chip was then functionally maximized by converting a 2D intestine model to a 3D intestine model. Bases with arrayed protrusions of various shapes were biologically fabricated to mimic the shape and function of the real intestine in vitro. To realize these protrusions, techniques have been developed to fabricate villi-like scaffolds based on various proteins (ECM, collagen, Matrigel, and so on) or other biomaterials to closely mimic real gut protrusions. Different bio–microfabrication technologies such as micro-molding, micromachining, soft lithography, bio-printing, and stamping have been employed. However, this 3D formation of villi-mimicking protrusions tended to reduce utilization by increasing the complexity of the fabrication process. Although 3D intestine chips have been successfully fabricated by hydrogel-based bio–microfabrication technology, limitations of the fabrication technology resulting from miniaturization for more precise intestine simulation still need to be overcome.

Overall, the gut chip used in this study, with the greatest ripple effect, is a microfluidic cell culture device with two parallel multilayer channels separated by an ECM-coated microporous membrane. This biomimetic device was developed by the Ingber group in 2012 and has been successfully used in 2D and 3D studies to simulate internal organs and internal circulation models such as the gut and lung in vitro [25,58,59,65,82,84,94,95,96,97,98]. In addition, other researchers have improved together by modifying and applying it to suit a number of studies due to the advantages of convenience of chip manufacturing and efficiency of experiments. This leading research leads the development of chip fabrication technology and biomedical technology, and it is believed that a new paradigm of convergence research can be pioneered in the near future.

## Figures and Tables

**Table 1:** Bio–microfabrication techniques for two-dimensional gut chips.

Cell Culture Base Type	Chemical Stimulus for 2D Formation	Physical Stimulus for 2D Formation	Microfabrication Technique	Application	Cell Type	Ref. (Year)
Porous Six-well plate 1.0 µm inserts	Collagen		Photolithography; soft lithography	Assessment of intestinal absorption, hepatic metabolism, and bioactivity	Caco-2	[74] (2010)
Porous PDMS membrane with 10 µm pores	Collagen; Matrigel	Cyclic mechanical strain	Photolithography; soft lithography	Fabrication of gut chip inhabited by microbial flora for drug testing	Caco-2	[58] (2012)
Polyamide membrane with a pore size of 0.2 μm and a thickness of 115 μm	Collagen; agar	Homogeneous fluid shear distribution	Soft lithography	Study of the host–microbe interaction	Caco-2; enterocyte	[16] (2014)
Polyethylene–terephlalate microporous membrane	Collagen	Hydrodynamic shear stress	Photolithography; soft lithography	Pharmacokinetic study	Caco-2	[72] (2015)
Porous PDMS membrane with an array of circular holes	ECM	Shear stress; cyclic strain	Photolithography; soft lithography	Microengineering of the model of human intestinal inflammation and bacterial overgrowth	Caco-2	[59] (2016)
Polycarbonate membrane	Collagen		Computer numerically controlled milling; laser cutting	Proving of causal relationships between gastrointestinal microbiota and human diseases	Caco-2	[51] (2016)
Polyester transwell with 0.4 µm pores	Collagen	Fluid shear stress	Micromachining	Quantitative analysis of relevant phenomena; including drug fate and exposure	Caco-2; HT29-MTX	[52] (2017)
Polyester transwell with 0.4 µm pores		Fluid shear stress	Soft lithography	Reproduction of the first-pass metabolism	Caco-2	[64] (2017)
Porous polyester membrane with 0.4µm pores	Collagen		Micromachining; CO_2_ laser cutting; e-beam evaporating	Trans-epithelial electrical resistance measurement of human epithelial barrier function	Caco-2	[65] (2017)
OrganoPlate	Collagen	Fluidic shear stress		Study of in vivo epithelial barrier physiology	Caco-2	[75] (2017)
Polycarbonate nucleopore track-etch 0.4 μm pore size membrane	Collagen		Micromachining	Evaluation of enzyme activities of P450 1A1 and P450 3A4 and the levels of urea and albumin	Human intestinal myofibroblast	[68] (2018)
Polystyrene substrate	Collagen		Photolithography; soft lithography; micro-milling	Drug metabolism	Caco-2	[71] (2018)
Thiolene-coated teflon membrane	Collagen; Matrigel		Replica molding; micro-milling; soft lithography; metal deposition	Drug transport study	Caco-2	[53] (2018)
Porous polycarbonate membrane with 1.0 μm pore size	Collagen	Shear stress	Soft lithography; replica molding; milling	Accurate drug testing	Caco-2	[69] (2019)
Polyether membrane with 1.0 μm pore size	Collagen	Shear stress	Soft lithography; milling	Systems biology	Caco-2	[76] (2019)
Microporous PDMS membrane	Collagen; Matrigel	Fluid shear stress	Soft lithography; 3D printing	Study of intestinal metabolism, homeostasis, and immune regulation	Caco-2	[62] (2019)
Porous PDMS membrane		Fluid shear stress	Soft lithography	Study of host–microbial interaction	Caco-2	[57] (2020)
Hollow fiber membrane	Collagen	Shear stress	3D printing	Optimization of cell differentiation	Caco-2	[77] (2020)
Porous PDMS membrane containing 7 µm diameter pores	Collagen	Shear stress; cyclic strain	Soft lithography	Study of drug pharmacokinetics and personalized drug therapy	Caco-2	[63] (2020)
Porous teflon membrane with a pore size of 0.4 μm	Collagen		Photolithography; soft lithography	Visualization of bacterial colonization and cellular layers	Caco-2	[73] (2020)
PDMS membrane with a thickness of 2 μm and 5 μm pore size	Collagen		Replica molding; micro-milling; soft lithography	Measurement of the barrier function	Caco-2	[55] (2021)
Polyester membranes with 0.4 µm pore size		Shear stress	3D printing; soft lithography	Study of transport across epithelial and endothelial barriers	Caco-2	[66] (2021)
Porous PDMS membrane with 7 µm pore size	Collagen	Cyclical stress	Soft lithography; fabricated by Emulate Bio	Treatment of phenylketonuria	Caco-2; HT-29 MTX	[60,61] (2021)
Glass slide	ECM	Fluid shear stress	Soft lithography	Highly-personalized medicine and in vivo phage applications	HT-29 MTX; gut epithelial cell	[78] (2022)
Microchannel	Collagen; Matrigel	Shear stress	Photolithography; soft lithography; metal deposition	Study of the microbiome to intestinal inflammation	Human Caco-2	[79] (2022)
Polycarbonate membranes with pore size of 8 μm	Collagen; Matrigel		Soft lithography	Exploration of host–microorganism interaction and enteritis treatment	Human Caco-2	[70] (2022)
Porous PDMS membrane with 12 µm pore size	Collagen; Matrigel		Photolithography; soft lithography	Development of re-useable SU-8 molds	Human Caco-2	[56] (2023)
Porous polyester membrane with a pore size of 0.4 μm		Shear stress	Photolithography; soft lithography	Intestinal absorption study	Human Caco-2	[67] (2023)
Teflon membrane (Emotion) with 5 μm diameter of the pore		Fluid shear stress; cyclic mechanical strain	Soft lithography	Exploration of the transport mechanism of Hg (II)	Human Caco-2	[54] (2023)

**Table 2:** Bio–microfabrication techniques for three-dimensional gut chip.

Cell Culture Base Type	Chemical Stimulus for 3D Formation	Physical Stimulus for 3D Formation	Microfabrication Technique	Application	Cell Type	Ref. (Year)
Collagen and Matrigel-coated polyester porous membrane with 0.4 µm pore	Collagen; Matrigel	Cyclic mechanical strain	Photolithography; soft lithography	Study on intestinal physiology and digestive diseases	Human Caco-2	[82] (2013)
Polyester Track Etched (PETE) membrane with 1 μm pores		Pulsatile fluid flow	Soft lithography	Long-term co-culture of human intestine, liver, skin and kidney equivalents	Primary human small intestinal epithelial cell	[40] (2015)
Extracellular matrix-coated PDMS membrane containing an array of pores (10 μm in diameter)	Extracellular matrix	Peristalsis-associated mechanical deformation; shear stress	Photolithography; soft lithography	Study of probiotic and antibiotic therapies	Human Caco-2	[59] (2016)
Collagen scaffold	Collagen	Fluidic shear stress	Photolithography; soft lithography; replica molding	Study of physiological relevance	Human Caco-2	[83] (2017)
Collagen and Matrigel-coated PDMS porous PDMS membrane with pores of 10 μm in diameter)	Collagen; Matrigel	Apical flow; cyclic mechanical strain	Soft lithography	Analysis of human enterovirus infection; replication and infectious virus production	Human Caco-2	[84] (2017)
Collagen coated rounded-top posts	Collagen		Micro-molding; PDMS stamping; photolithography	Generation of a polarized crypt-villus architecture of human small intestinal epithelium	Human villi and crypts	[85] (2017)
PMMA with holed grid	Collagen	Optimized air-liquid interface culture	Micro-milling; micro-molding	Induction of polarized crypt-villus structure of human small intestinal epithelium	Human intestinal subepithelial myofibroblast; human Caco-2	[86] (2018)
Collagen scaffold	Collagen		Stamping; micro-molding	Application of chemical gradients across a shaped epithelial monolayer	Human colonic crypt; human Caco-2	[87] (2018)
Matrigel-coated PDMS membrane	Matrigel	Fluidic flow	Photolithography; soft lithography	Incorporating human intestinal organoids into small micro-engineered chips	Human intestinal organoid derived intestinal epithelial or Caco2	[88] (2018)
Villi-like PEGDA and acrylic acid scaffold array	Collagen		Photolithography; stencil	Integration with standard cell culture platforms	Caco2	[89] (2019)
Hydrogel scaffolds	Collagen/SIS		bioprinting	Realization of efficient digestion and absorption	Caco2	[80] (2020)
Microchannel coated with extracellular matrix (ECM) proteins	Matrigel; collagen	Multiaxial deformations in a cycle of stretching motion	Soft lithography	Studying the host–gut microbiome crosstalk associated with gastrointestinal diseases	Caco-2; organoid-derived epithelial cell	[81] (2020)
Matrigel microchannel	Matrigel	Dynamic flow; shear stress	Soft lithography	Realization of intestinal cells morphology and polarization	Caco-2	[90] (2021)
Silk sponge scaffold	Silk fibroin protein and collagen		Micro-molding; soft lithography	Fabrication of bioengineering functional human intestinal epithelium	Caco-2; HT29-MTX	[91] (2022)
ECM-coated PDMS porous membrane	ECM	Shear stress, mechanical motions	Soft lithography	Establishment of 3D in vitro morphogenesis	Caco-2	[92] (2022)
Porous membrane with 20 μm thick; 5 μm diameter of the pore		Mechanical stretch	Soft lithography	Exploration of the transport mechanism of Hg (II)	Caco-2	[54] (2023)
Silicon wafer containing post arrays of circular pillars (10 μm diameter × 20 μm height with 25 μm spacing)	Matrigel, collagen	fluid shear stress	Soft lithography	Study of the contribution of Bifidobacterium bifidum to inflammatory bowel disease	Caco-2	[93] (2023)

## Data Availability

Not applicable.
